# State of the art in advanced endoscopic imaging for the detection and evaluation of dysplasia and early cancer of the gastrointestinal tract

**DOI:** 10.2147/CEG.S58157

**Published:** 2014-05-13

**Authors:** Sergio Coda, Andrew V Thillainayagam

**Affiliations:** 1 Section of Gastroenterology and Hepatology, Department of Medicine and Photonics Group, Department of Physics, Imperial College London, London, UK; 2 Endoscopy Unit, Charing Cross Hospital, Imperial College Healthcare NHS Trust, London, UK

**Keywords:** image-enhanced endoscopy, narrowband imaging, autofluorescence imaging, confocal laser endomicroscopy, fluorescence lifetime imaging

## Abstract

Ideally, endoscopists should be able to detect, characterize, and confirm the nature of a lesion at the bedside, minimizing uncertainties and targeting biopsies and resections only where necessary. However, under conventional white-light inspection – at present, the sole established technique available to most of humanity – premalignant conditions and early cancers can frequently escape detection. In recent years, a range of innovative techniques have entered the endoscopic arena due to their ability to enhance the contrast of diseased tissue regions beyond what is inherently possible with standard white-light endoscopy equipment. The aim of this review is to provide an overview of the state-of-the-art advanced endoscopic imaging techniques available for clinical use that are impacting the way precancerous and neoplastic lesions of the gastrointestinal tract are currently detected and characterized at endoscopy. The basic instrumentation and the physics behind each method, followed by the most influential clinical experience, are described. High-definition endoscopy, with or without optical magnification, has contributed to higher detection rates compared with white-light endoscopy alone and has now replaced ordinary equipment in daily practice. Contrast-enhancement techniques, whether dye-based or computed, have been combined with white-light endoscopy to further improve its accuracy, but histology is still required to clarify the diagnosis. Optical microscopy techniques such as confocal laser endomicroscopy and endocytoscopy enable in vivo histology during endoscopy; however, although of invaluable assistance for tissue characterization, they have not yet made transition between research and clinical use. It is still unknown which approach or combination of techniques offers the best potential. The optimal method will entail the ability to survey wide areas of tissue in concert with the ability to obtain the degree of detailed information provided by microscopic techniques. In this respect, the challenging combination of autofluorescence imaging and confocal endomicroscopy seems promising, and further research is awaited.

## Introduction

Cancer of the gastrointestinal (GI) tract is the leading cause of cancer death worldwide.^1^ Prevention is based on early endoscopic detection of potentially curable cancers or precursor conditions such as dysplasia, which have a significant risk of progression to malignancy.^2^ Thus, early diagnosis is the critical goal, since it allows endoscopic or surgical intervention on a localized disease without lymph node involvement, which greatly improves patient survival.

However, easy and accurate detection of dysplasia, early detection of malignancies and accurate discrimination of inflammatory disease from neoplasia remain the main challenges in GI endoscopy. There are at least four main domains where the clinical needs are still unmet. For instance, in Barrett’s esophagus (BE), up to 40% of high-grade dysplasia (HGD) was found to be associated with inconspicuous synchronous occult foci of adenocarcinoma after esophagectomy.^3^,^4^ Standard white-light endoscopy (WLE) identifies BE and obvious mucosal abnormalities (eg, nodules, and raised and depressed areas) but cannot distinguish intestinal metaplasia from dysplasia or other types of metaplasia (cardiac or oxyntic) not at risk of malignancy. Moreover, the current standard method for detecting dysplasia in patients with BE is a random four-quadrant biopsy protocol (Seattle protocol), but dysplasia can easily be missed, as only a small fraction of the Barrett’s segment (less than 3.5% of the total surface of a 2 cm long segment) is sampled.^5^

Detection of early gastric cancer is still a gray area in clinical endoscopy due to the minute changes such as faint mucosal irregularities or discoloration, which can be easily overlooked. For instance, apart from Japan, only 10% of gastric cancers are detected at an early stage in most of the world.^6^ Although chromoendoscopy is increasingly employed, the identification of early cancer remains poor unless an obvious abnormality such as an ulcer or mass is found.

Adenoma miss rates of up to 25% during colonoscopy have been reported.^7^–^9^ Interval cancers have also been found in patients with a history of adenomatous polyps, despite regular colonoscopy surveillance.^10^,^11^ Adenomas usually originate as diminutive polyps. However, only two-thirds of colorectal cancer (CRC) seems to develop through the stage of adenoma, the other third growing de novo from normal epithelium.^12^ Aberrant crypt foci, defined as colonic crypts with a larger diameter and a thicker epithelium than normal mucosa, have been proposed as one of the earliest stages of malignant transformation. However, these changes are too subtle to be visualized with a standard endoscope.^13^ Similarly, detection of flat polyps, which are characterized by a high malignant potential compared with sessile and pedunculated polyps,^14^,^15^ can be extremely difficult because of the less well-defined subtle findings.

Lastly, dysplasia and malignant transformation represent the most important complication in patients with inflammatory bowel disease (IBD). At present, annual endoscopic surveillance is recommended after 8–10 years of disease. In patients with ulcerative colitis, if no lesions are observed, four biopsy specimens are taken randomly at every 10 cm between the rectum and the cecum for a total of 40–50 biopsies per colonoscopy.^16^ However, even this massive sampling regimen examines less than 1% of the total colonic mucosa surface. In addition, the natural history of dysplasia in the context of IBD is poorly understood. Dysplasia in IBD can be flat (endoscopically invisible) or elevated (endoscopically detectable).^17^ Elevated lesions are referred to as dysplasia-associated lesions or masses (DALMs) and are broadly separated into adenoma-like and non-adenoma-like depending on whether or not they resemble sporadic adenomas unrelated to IBD. It is still unclear whether all elevated dysplastic lesions begin as flat endoscopically invisible dysplasia, and it is extremely difficult, if not impossible, to discriminate dysplasia from regenerative or inflammatory changes, both in presence or absence of a visible lesion (ie, pseudopolyps, DALM, and raised or depressed areas of mucosa). Another major problem during surveillance for cancer in patients with IBD is the histological finding of dysplasia in random biopsies of diffusely inflamed mucosa as well as of macroscopically normal mucosa, and not in those targeted to a visible lesion.^17^

In recent years, a range of innovative techniques have been developed and adapted for clinical endoscopy with the aim of addressing these unmet clinical needs, providing a “red flag” technique, and perhaps ultimately revealing lesions that are still invisible under conventional WLE. Some of these methods enhance and optimize the inherent contrast available, while others offer the possibility of assessing the tissue structure in real-time at a microscopic level.

Commercially available techniques include high-definition endoscopy (HDE),^18^–^20^ magnifying chromoendoscopy,^21^ narrow-band imaging (NBI),^22^ Fuji intelligent chromoendoscopy (FICE),^23^ i-Scan (Pentax; Hoya Corporation, Tokyo, Japan),^24^ autofluorescence imaging (AFI),^25^ confocal laser endomicroscopy (CLE), whether by integrated technology (endoscope-based CLE [eCLE])^26^ or through-the-scope probe-based CLE (pCLE),^27^ and endocytoscopy (EC).^28^ Among these techniques, endoscopic confocal microscopy has gained prominence since 2004, when the first in vivo study appeared on the clinical scene.^26^ For clinical applications in gastroenterology, pCLE, an evolution and miniaturization of CLE, was subsequently launched in 2007,^27^,^29^ and highly promising results have been reported since then.^27^,^30^–^34^ Many other potential applications are yet to be validated, and research to make CLE “label-free” is currently in progress.^35^

**Figure 1:**
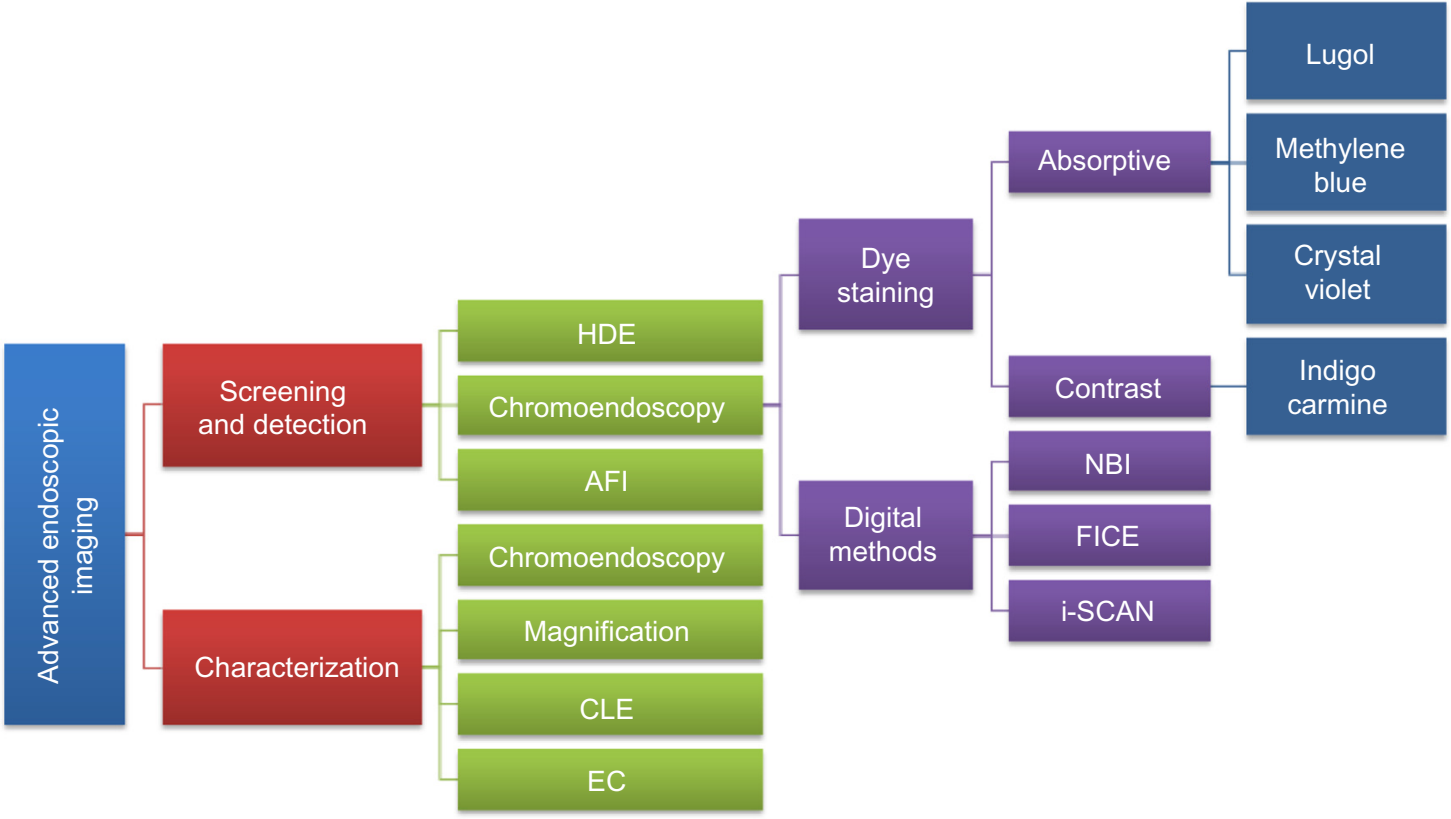
Goal-oriented classification of image-enhancement, magnifying, and microscopic techniques currently available and approved for clinical use. **Note:** i-Scan is manufactured by Pentax. **Abbreviations:** AFI, autofluorescence imaging; CLE, confocal laser endomicroscopy; EC, endocytoscopy; FICE, Fuji intelligent chromoendoscopy; HDE, high-definition endoscopy; NBI, narrow-band imaging.

A consensus methodological classification of endoscopic imaging proposed by Tajiri and Niwa^36^ in 2008 divides the existing techniques into five major categories: 1) conventional (WLE); 2) image-enhancement (further subdivided into digital, optical-digital, and chromoendoscopy methods); 3) magnifying (optical and digital); 4) microscopic (CLE and EC); and 5) tomographic (endoscopic ultrasonography and optical coherence tomography). Figure 1 provides an overview together with a goal-oriented classification of the currently available image-enhancement, magnifying, and microscopic techniques (categories 2, 3, and 4, respectively) approved for clinical use in GI endoscopy.

This review is based on a comprehensive MEDLINE^®^ search of studies published since 1995, using the terms “dysplasia,” “early gastrointestinal cancer,” “magnification,” “high definition endoscopy (HDE),” “narrow band imaging (NBI),” “autofluorescence imaging (AFI),” “Fuji intelligent chromoendoscopy (FICE),” “i-Scan,” “confocal laser endomicroscopy (CLE),” and “endocytoscopy (EC).” Only extensively referenced studies describing the use of clinically approved commercially available techniques, as defined by the Tajiri and Niwa classification of endoscopic imaging,^36^ and investigating the detection/evaluation of GI dysplasia and early cancers, were included. Both authors contributed equally to the search and selection of the studies, design of the review, discussion and interpretation of results, and critical revision, also reflecting personal experience and expert opinions. The aim of this review is to provide a concise and comprehensive synopsis of the latest advanced imaging techniques that are impacting the way precancerous and neoplastic lesions of the GI tract are currently detected and characterized at endoscopy. It embraces both: 1) evaluation of all available modalities, those in day-to-day use as well as those under experiment; and 2) identification of directions for future investigation/research. For each technique, the fundamental principles and instrument specifications are summarized as well as their clinical application and potential roles as tools for the endoscopist. Particular emphasis is given to the currently available techniques that are still research based (eg, AFI and CLE) and need consolidation, rather than to those closer and already implemented in the clinical practice (eg, HDE, chromoendoscopy, and virtual chromoendoscopy). One role envisaged is a “red flag” guide to potential areas of malignancy, reducing time and the number of diagnostic biopsies taken. In this respect, the potential combination of current techniques as well as the integration of future potential clinical optical imaging modalities, such as fluorescence lifetime imaging microscopy (FLIM), is anticipated. This information may help guide future studies in this field and assist in developing more accurate, lower cost, and faster instrumentation.

## HDE, chromoendoscopy, and magnification endoscopy

As in modern television technology, high-definition video endoscopes utilize charge-coupled devices (CCDs) with over a million pixels compared with older CCDs with an average of 300,000.

Magnifying endoscopes have a lens system built into the distal tip of the instrument. This zoom can be used to magnify areas of GI mucosa from ×6 to ×150. The main difficulty when using this endoscope is to keep the instrument still and maintain the interface between tissue and tip of the instrument constant for accurate focusing. Particularly in upper GI endoscopy, breathing and peristalsis require continuous adjustments.

**Figure 2:**
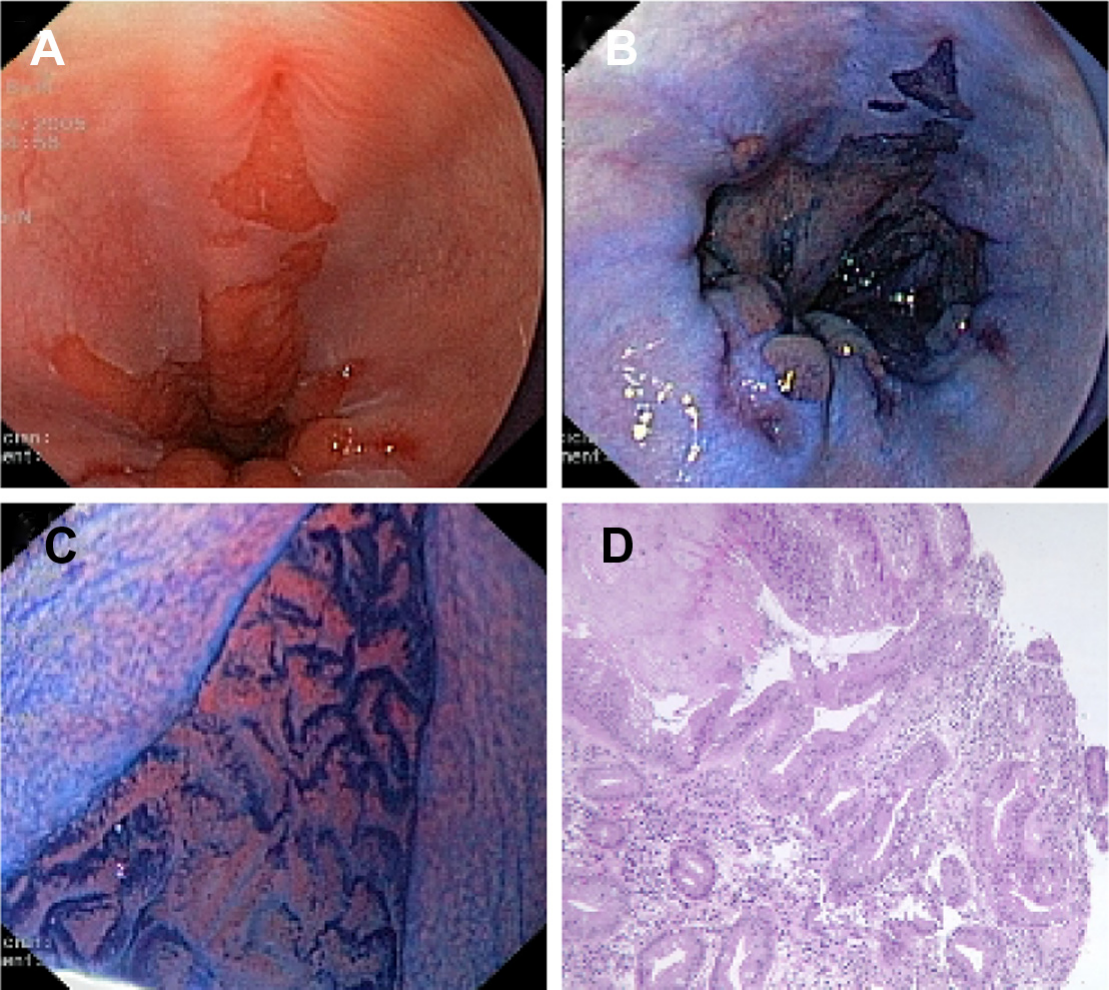
(**A**) Conventional endoscopic view of Barrett’s esophagus with concomitant esophagitis. (**B**) Positive staining of Barrett’s epithelium after absorption chromoendoscopy with methylene blue dye solution (1%, 10 mL). (**C**) Villous cerebroid pits with finger-like projections seen with magnification endoscopy (pattern 5 according to Endo’s classification). (**D**) Histological section of (**C**) showing intestinal metaplasia with glands of different size and shape and numerous goblet cells. **Note:** Images provided courtesy of Dr Sergio Coda and Professor Paolo Trentino, University of Rome “La Sapienza,” Italy.

Magnification is often combined with the use of vital dyes (chromoendoscopy) to enhance the contrast in the surface (Figure 2).

## Clinical experience

### Upper GI tract

Lugol’s vital staining is the most commonly used method to enhance the detection of esophageal squamous dysplasia and early squamous cell carcinoma in high-risk populations, and variable sensitivity (91%–100%) and specificity (40%–95%) have been reported.^37^–^39^

In BE, methylene blue is undoubtedly the preferred dye to enhance the detection of dysplasia and early cancer, although its value is still controversial due to the reported wide range of sensitivities (32%–98%) and specificities (23%–100%),^40^ high level of interobserver variability,^41^ and inconsistent rates of increased detection.^42^–^46^

Using magnifying endoscopy and methylene blue, Endo et al^47^ proposed a classification of five esophageal pit patterns to distinguish metaplastic epithelium from gastric phenotypes. Based on correlation with histology and mucin phenotypes, types 4 and 5 were found to be highly related to the presence of intestinal metaplasia.

Clinical experience with other staining methods in BE remains limited, and their role has not yet been established. Among others, use of indigo carmine in combination with enhanced magnification endoscopy has been found useful in distinguishing non-dysplastic from dysplastic pit patterns.^48^

Four different mucosal pit patterns were identified using magnifying endoscopy with acetic acid in patients with short-segment BE without dysplasia. In this study, types 3 and 4 were shown to contain intestinal metaplasia in 87% and 100%, respectively, of biopsy specimens.^49^

Methylene blue staining with magnifying endoscopy has also been applied to detect gastric intestinal metaplasia and dysplasia with 84% and 83% accuracy, respectively. In this study, specific mucosal patterns were identified and classified into three groups: 1) non-metaplastic, non-dysplastic mucosa; 2) metaplastic mucosa; and 3) dysplastic mucosa.^50^ However, in some cases, it is practically difficult to achieve reliable and clear pattern identification (and interobserver agreement) due to uneven spreading of the dye and heterogeneous staining of the gastric mucosa.

### Lower GI tract

By using magnifying endoscopes and indigo-carmine dye spraying, Kudo et al^21^ proposed a classification of five different polyp pit patterns associated with a percentage of correlation with the histology. This classification was meant to predict an increasing risk of neoplastic changes and assist clinicians in their therapeutic strategy planning. Kato et al,^51^ in one of the largest studies comparing these pit patterns with histology (3,438 lesions) found an accuracy of 75% for non-neoplastic lesions, 94% for adenomas, and 85% for carcinomas.

Chromoendoscopy is gaining more importance, as some of the colon cancers may not develop via the classical sequence adenoma-cancer but have only the transient phase of a small flat adenoma (de novo sequence).^52^

In a multicenter trial with 1,000 colonoscopies, the number of adenomas detected was almost 40% higher with the combination of magnification and chromoendoscopy compared with standard colonoscopy.^53^

In contrast, in another large multicenter study from France including 203 patients, no difference was found in the overall adenoma detection rate between standard-resolution (≤410,000 pixels) WLE and high-resolution (850,000 pixels) WLE coupled with pan-colonic chromoendoscopy with indigo carmine (49.5% versus 59.4%, respectively). However, interestingly, high-resolution chromoendoscopy yielded a higher number of hyperplastic polyps and flat adenomas <5 mm than pan-colonic chromoendoscopy with indigo carmine.^54^

HDE, with or without optical magnification, has now replaced ordinary equipment in daily practice, and its combination with dye staining is expected to become the standard of care for selected patients who need surveillance (eg, CRC, IBD, and BE). However, many aspects of this technique still need to be standardized (eg, the amount of dye to spray, the use of a spraying catheter, the power to be applied when spraying, which in turn determines the more or less homogeneous coloration of the target area). Nonetheless, although the effectiveness of chromoendoscopy has been shown,^55^,^56^ staining of the entire colon remains a time-consuming procedure.

## Virtual chromoendoscopy

### NBI

**Figure 3:**
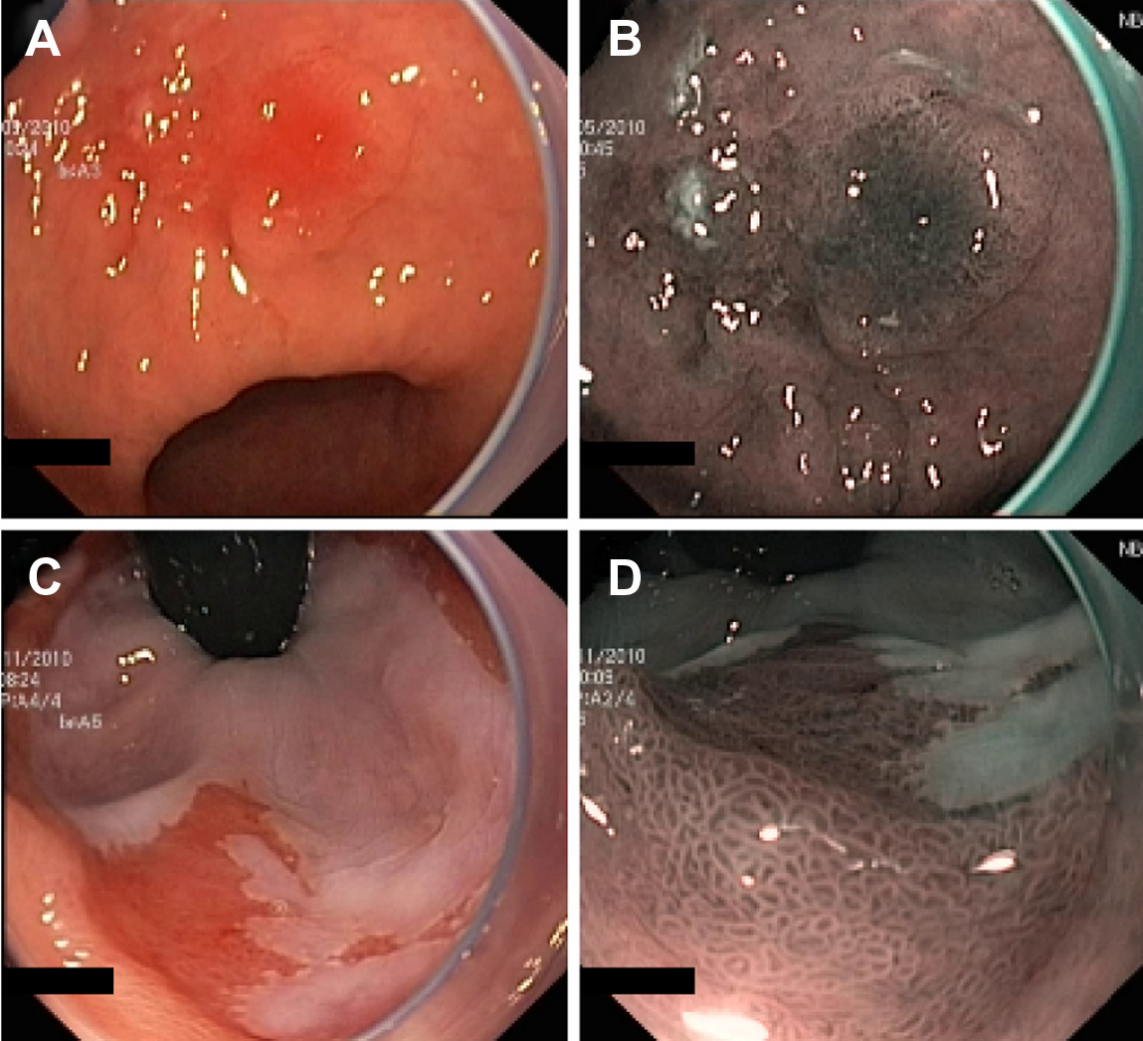
Example images of areas of suspected early cancers of the gastric antrum (**A** and **B**) and cardia (**C** and **D**), imaged using standard WLE (**A** and **C**) and NBI (**B** and **D**) to demonstrate the contrast enhancement provided by NBI. **Note:** Images provided courtesy of Professor Paolo Trentino, University of Rome “La Sapienza,” Italy. **Abbreviations:** NBI, narrow-band imaging; WLE, white-light endoscopy.

NBI (Olympus Corporation, Tokyo, Japan) is a digital filter technique that improves the visibility of capillaries, veins, and other subtle tissue structures by optimizing the absorbance and scattering of light without the use of contrast or absorptive dyes. Example images of NBI in comparison with conventional WLE are shown in Figure 3.

**Figure 4:**
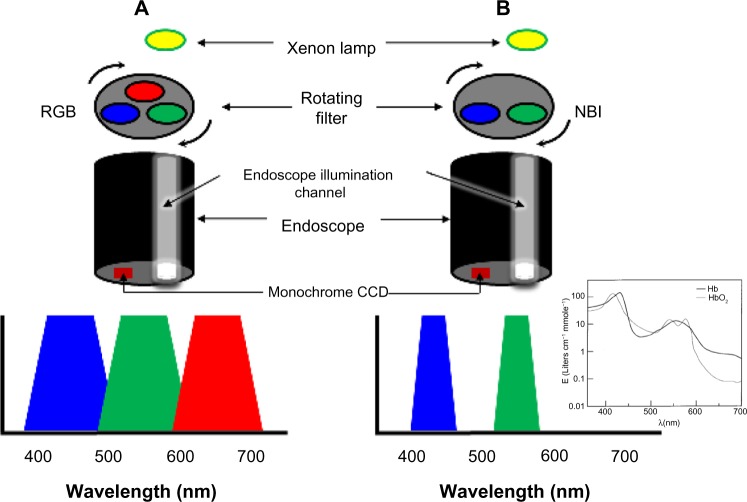
Schematic diagram showing the difference between a standard RGB filter (**A**) and the NBI filter (**B**). **Notes:** Compared with the full range of white-light illumination, the filtered light penetrates the tissue less, highlighting the superficial details of the mucosa. Additionally, the filtered centered wavelengths fall within hemoglobin absorption bands (inset of **B**), and this leads to a higher contrast for vascular structures. The inset of B is reproduced from Zonios G, Perelman LT, Backman V, Manoharan R, Fitzmaurice M, Van Dam J, Feld MS. Diffuse reflectance spectroscopy of human adenomatous colon polyps in vivo. *Appl Opt*. 1999;38(31):6628–6637.^57^ Copyright © 1999 Optical Society of America. **Abbreviations:** CCD, charge-coupled device; NBI, narrow-band imaging; RGB, red, green, and blue.

This system adapts the conventional additive RGB (red, green, and blue) color model of a conventional electronic endoscope with an ordinary light source (Figure 4A) and through the application of narrow-band filters to enhance the tissue microvascular architecture (Figure 4B).

NBI uses two bands of light, at 415 nm (blue) and at 540 nm (green). Narrow-band blue light preferably displays superficial capillary networks, while green light selectively displays deeper subepithelial vessels, and when combined, they offer an extremely high contrast image of the tissue surface. Commercial NBI systems apply image processing so as to display capillaries on the surface in brown, and veins in the subsurface in cyan.

NBI is based upon the phenomenon that incident light with longer wavelength undergoes less scattering and thus penetrates further into the tissue. Blue light penetrates only superficially, whereas red light penetrates into deeper layers. The choice of the two bands of light at 415 and 540 nm is determined by the peaks of absorption of hemoglobin (maxima at 415, 542, and 577 nm for oxyhemoglobin, and at 430 and 555 nm for deoxyhemoglobin^57^ (see inset of Figure 4B), thus the centered wavelengths resulting from the narrow-band filtering lead to a higher contrast for vascular structures.

The first prototype of the NBI system was developed by Gono et al in Japan.^22^,^58^ White light from a xenon lamp is conveyed through a rotary RGB filter that separates the white light into the colors red, green, and blue, which are used to sequentially illuminate the mucosa via the illumination channel of the endoscope (Figure 4). The red, green, and blue reflected light is detected sequentially by a monochromatic CCD placed at the tip of the endoscope, and the three images are integrated into a single color image by the video processor. In addition to the conventional RGB filters for WLE, the NBI system has filters of which the band-pass ranges have been narrowed and the relative contribution of blue light has been increased.

Compared with chromoendoscopy, NBI offers the advantage of providing contrast without the use of dyes, but the general endoscopists’ confidence with this system is still under debate. One aspect of NBI is that images are less bright than those of WLE; therefore, it is often difficult to observe large areas from a distant view as the image quality significantly degrades with distance. This makes it more suited to detailed mucosal inspection rather than screening.

#### Clinical experience

Various studies have reported on the usefulness of NBI for enhanced detection of dysplasia and neoplasia, both in the upper and in the lower GI tract. The results are inconsistent and suggest that the clinical utility of NBI in day-to-day practice is limited.

#### Upper GI tract

NBI as an adjunct technique to HDE, has shown a comparable performance to chromoendoscopy when applied in a randomized cross-over study of 28 patients with BE, and does not improve the overall sensitivity for identifying patients with HGD or early cancer.^59^ In a study with 56 patients with BE undergoing endoscopic surveillance for previously detected dysplasia, NBI detected significantly more dysplasia, and with recourse to fewer targeted biopsies, compared with WLE alone.^60^

In a recent study by Sharma et al^61^ in 123 patients with BE with mean circumferential and maximal extents of 1.8 and 3.6 cm, respectively, NBI revealed more areas of dysplasia in patients with fewer biopsies when compared with HDE plus Seattle protocol.

#### Lower GI tract

Early prospective comparative studies have reported higher diagnostic accuracies than WLE in distinguishing neoplastic from non-neoplastic polyps based on vascular and pit pattern characteristics. However, three randomized controlled trials failed to show an increased adenoma detection rate when comparing NBI with WLE.^18^,^62^

The vascular pattern intensity, a measure of microvascular density, was proposed by East et al^63^,^64^ as a new classification parameter to discriminate neoplastic from non-neoplastic polyps with NBI.

In a study of 62 patients with hereditary nonpolyposis CRC, the adenoma detection rate was almost doubled when a second inspection with NBI was performed compared with standard colonoscopy alone.^65^

Using microvascular networks as a marker of neoplasia in a variety of organs including colon, esophagus, and lungs, a meta-analysis of eleven studies comparing NBI-based diagnoses of neoplasia with histopathology revealed a high diagnostic precision for detection of neoplasia, with an overall sensitivity and specificity of 94% and 83%, respectively.^66^ In contrast, other studies have failed to demonstrate an improved detection of neoplastic polyps^18^,^19^,^62^ and dysplasia in patients with longstanding ulcerative colitis.^67^

Nowadays, the NICE (NBI international colorectal endoscopic) classification is the most extensively validated classification used worldwide, by which colorectal tumors can be simply classified, with or without magnification, into three types based on the color of the lesion, the microvascular architecture, and the surface pattern. Type 1 lesions are considered hyperplastic. Type 2 lesions include all adenomas (with low-grade dysplasia or HGD), carcinomas-in-situ or intramucosal carcinomas, and some lesions with superficial submucosal invasion. Invasive carcinomas with deep submucosal invasion have type 3 appearance.^68^

### FICE and i-Scan

Along with NBI, the most recent development in virtual chromoendoscopy is the computed post-processing spectral estimation technology invented by Miyake et al^69^,^70^ and introduced with the FICE system by Fujinon Corporation (Saitama, Japan). A similar system was released in April 2007 by Pentax Corporation, the so called i-Scan technology.

In contrast to NBI, where the illumination light is filtered to achieve contrast, in FICE and i-Scan the contrast is obtained after the illumination has reached the tissue by processing the spectral reflectance captured by the CCD.

The FICE system is based on computed spectral estimation of photons reflected from an ordinary white-light image. In this way, white-light images are sent to a spectral estimation matrix processing circuit and reconstructed in three distinct virtual single-wavelength images: red, green, and blue. By combining these three single wavelength images, a resultant FICE-enhanced color image is then obtained.

i-Scan technology is based on digital filter enhancement and software manipulation of color tone, sharpness, and contrast of high-definition images (1.25 million pixels). i-Scan can be used in three modes depending on the level of enhancement desired: 1) surface enhancement for easier demarcation of edges and flat lesions by enhancing light-to-dark contrast; 2) contrast enhancement for better identification of depressed lesions by enhancing areas of low intensity; and 3) tone enhancement for improved mucosal structure assessment by increasing the illumination and emphasis on vascular features.

#### Clinical experience

In patients with BE complicated by suspected HGD or early cancer, FICE shows similar sensitivity to acetic acid chromoendoscopy (87% for both techniques on a “per lesion” basis, and 92% versus 83% on a “per patient” analysis for FICE and chromoendoscopy, respectively).^71^ An increase in sensitivity is observed by combining targeted biopsies plus standard four-quadrant random biopsies (88% and 96% in the “per patient” analysis for FICE and chromoendoscopy, respectively).

When compared with WLE, transnasal FICE provides clearer delineation of the esophagogastric junction by identifying the palisade vessels – a reliable, but often invisible to WLE, anatomical marker of esophageal origin for measuring the extension of Barrett mucosa with accuracy.^72^ However, detection of dysplasia is not attempted due to the limited field of view (FOV) of the endoscopes used.

In a study by Neumann et al,^73^ double-balloon enteroscopy-assisted FICE failed to improve the detection or delineation of ulcers and erosions in three patients with Crohn’s disease but was found to be useful for the characterization of adenomatous polyps and angiodysplasias of the small bowel.

Hoffman et al^24^ reported a significantly increased detection rate of small (≤5 mm) adenomas for i-Scan when compared with WLE alone (11 versus 5, respectively). The inspection was conducted in the last 30 cm of the colon during the withdrawal phase of colonoscopy. Interestingly, the same detection rate was found for both conventional chromoendoscopy with methylene blue and i-Scan, although chromoendoscopy was considerably more time-consuming than i-Scan (13 versus 5 minutes). In contrast, in a recent study by Basford et al^74^ involving 209 polyps in 84 patients, no significant differences between HDE and i-Scan were observed in overall diagnostic accuracy of polyps <10 mm (93.3% versus 94.7%).

### AFI

AFI is a technique based on the principle that excitation of tissue with specific wavelength (eg, in the blue) leads to emission of a longer wavelength of light. In the GI tract, AFI detects subtle changes in the concentration of specific chemicals in tissue that have the ability to fluoresce when excited (endogenous fluorophores). Malignant transformation is associated with emission of relatively longer wavelengths of light (shift from green toward the red end of the spectrum).

Currently, the only commercially available AFI devices are RGB-based video endoscopes with trimodal (WLE + NBI + AFI) capability (Evis Lucera Spectrum; Olympus Medical Systems Corp, Tokyo, Japan). Two separate monochromatic CCDs are located at the tip of these endoscopes. One CCD is for high-definition WLE and NBI, and the other CCD is specific for AFI.

In AFI mode, blue light (390–470 nm) and green light (540–560 nm) is sequentially generated by a xenon lamp and conveyed through a rotating color filter wheel. An interference filter situated proximally to the AFI CCD blocks the blue light excitation but allows tissue autofluorescence (500–630 nm) and reflected green light to pass through.^75^

**Figure 5:**
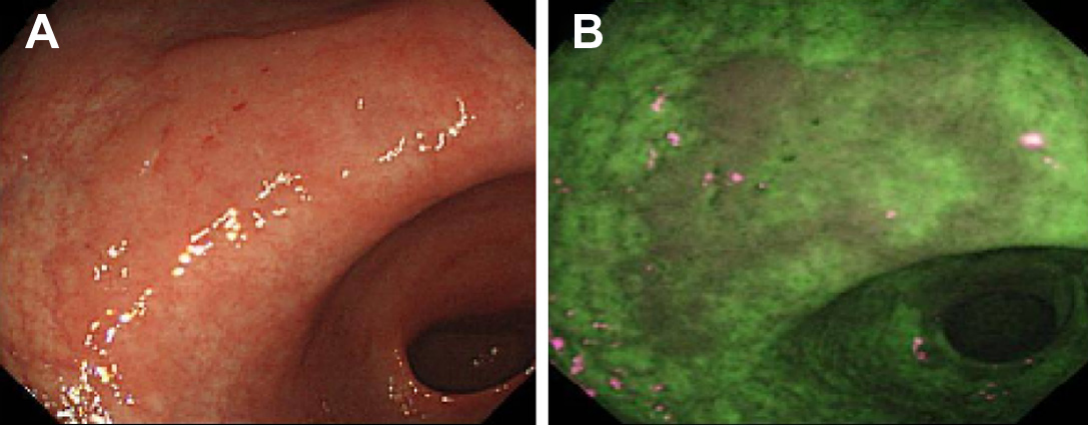
Example images of a suspected early cancer of the gastric antrum, imaged using standard WLE (**A**) and AFI (**B**), to demonstrate the contrast enhancement provided by AFI (Olympus Corporation, Tokyo, Japan). **Notes:** Images provided courtesy of Dr Chizu Yokoi, National Center for Global Health and Medicine, Tokyo, Japan. **Abbreviations:** AFI, autofluorescence imaging; WLE, white-light endoscopy.

The autofluorescence and green reflectance images are captured and integrated by the video processor into a single pseudocolor image, where normal mucosa typically appears green, and dysplastic or neoplastic tissue purple (Figure 5).f

However, the reason for the difference in fluorescence between normal and diseased tissue observed in current commercial AFI systems is still unclear, and intensity-based contrast is often not sufficiently specific.^75^–^77^

#### Clinical experience

##### Upper GI tract

A large multicenter randomized trial with patients affected by BE compared the diagnostic accuracy of surveillance with AFI-targeted biopsies plus Seattle protocol in comparison with the conventional Seattle protocol only. The investigators suggested that the AFI-guided biopsies improved the diagnostic accuracy for neoplasia in comparison with the conventional approach when using four quadrant biopsies. However, because of decreased sensitivity, they concluded that AFI alone was not suitable for replacing the standard Seattle protocol.^78^

More recently, AFI with blue-light excitation has been combined with high-definition WLE and NBI in a single endoscope with two CCDs. This technology is referred to as “trimodal imaging” and has been applied to identify inconspicuous Barrett’s neoplasia^79^ and assist endoscopic mucosal resection (EMR) of early neoplasia in BE.^80^ In an international multicenter study involving 84 patients with BE, the addition of AFI to high-resolution endoscopy increased the detection rate of early neoplasia within the Barrett segment, and additional viewing with NBI increased the overall specificity.^79^ However, as with AFI alone, the increased detection of dysplasia was only marginal (11%) and did not translate into a real benefit for the patients. Moreover, AFI alone was associated with a high false-positive rate (81%); therefore, the authors used NBI with optical magnification to further characterize areas detected by AFI, reducing the false-positive rate to 26% (at the expense of misclassifying two lesions as falsely negative).

Endoscopic molecular imaging using fluorescently labeled targeted peptides is being increasingly investigated for detection of inconspicuous dysplasia in BE. For example, glycans have been shown to be altered in GI cancers.^81^–^84^ Glycan changes can be detected using lectins, which have specific affinity for particular glycans. AFI and a fluorescently labeled lectin, wheat-germ (*Tritium vulgar*) agglutinin (WGA), have been used to detect changes in glycan expression on the epithelial cell surface associated with the transition from BE through dysplasia to adenocarcinoma.^85^ In particular, AFI has been used to image WGA in four esophagectomy specimens obtained immediately after surgery. The specimens were intubated from the proximal end, and both baseline WLE and AFI images were acquired. Fluorescein-labeled WGA was sprayed over the esophageal mucosa and imaged with 395–475 nm excitation. Specimens were then opened along their vertical axis and imaged using an IVIS^®^ camera (Caliper Life Sciences, Hopkinton, MA, USA) to enable quantification of fluorescence and registration with histology. A highly significant statistical correlation between WGA fluorescence and the degree of dysplasia was found (*P*=0.0002), with areas of HGD and cancer showing lower fluorescence intensity and WGA binding relative to areas of non-dysplastic BE and normal esophageal mucosa.

Kim et al^86^ performed preoperative AFI in 20 patients with early gastric cancers and then compared the endoscopic characteristics with histology after endoscopic submucosal dissection. Categorization of AFI images of gastric cancers into four patterns proved to be useful to delineate the dissection margins in the majority of cases.

##### Lower GI tract

A large body of literature exists on the use of autofluorescence to investigate colonic polyps and adenocarcinomas. Typically, colonic neoplasms have shown decreased autofluorescence intensity compared with that of normal colon. This can be attributed to the decrease with neoplasia of mucosal collagen, which is the dominant fluorophore, as a consequence of the enlargement of crypts that progressively displace the lamina propria. In addition, the submucosal contribution to the fluorescence in adenomatous tissue is reduced compared with that in normal colon due to the increased thickness of the polyp and absorption by hemoglobin, as a result of increased intra-tumoral microvessel density.

In a randomized trial on 100 patients undergoing screening colonoscopy, AFI was not able to significantly improve the diagnostic accuracy, with adenoma miss-rates similar to WLE.^87^

In a multicenter prospective randomized controlled study, the use of trimodal imaging did not improve the detection rate for adenomas compared with standard endoscopy. NBI and AFI showed very little accuracy and specificity in differentiating adenomas from non-adenomas (63% and 75% versus 37% and 62%, respectively).^88^ Also, differentiation of adenomas from hyperplastic polyps by using trimodal imaging in patients with hyperplastic polyposis was unsatisfactory.^89^

Another study from Japan showed that the florescence intensity of images acquired using an AFI system is inversely proportional to the degree of dysplasia in colonic adenomas.^90^ This study suggested that the dysplastic changes, including an increased number and density of cells and crypts, and the enlargement of nuclei and crypts, might somehow alter the tissue permeability and thus jeopardize the way back to the surface of the emitted fluorescence.

Since the assessment of AFI images is strictly dependent on color presented to the clinician by the image processing system used, quantifying the intensity of the color “magenta” by calculating a fluorescence index has been proposed as a method to discriminate lymphomas from reactive lymphoid hyperplasia,^91^ and to evaluate the degree of dysplasia in colonic neoplasms.^90^

### CLE

Confocal microscopy was invented by Minsky in the 1950s.^92^,^93^ Since its conception and development, confocal microscopy has been extensively used in biology and medicine for imaging living intact tissues without having to physically cut up and prepare thin sections as in histology.

**Figure 6:**
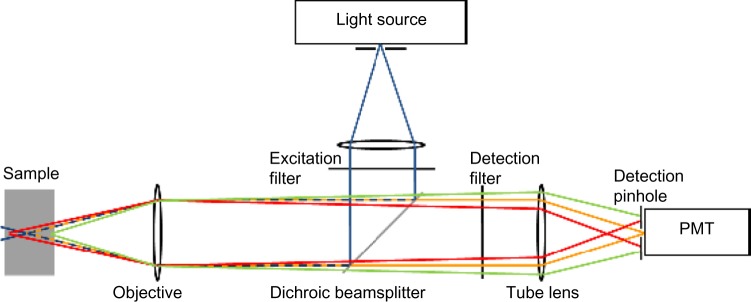
Schematic diagram of confocal microscopy principles. **Notes:** The blue rays (pre- and post-objective and excitation filter) indicate the laser illumination delivered to the tissue sample. The fluorescence emitted from a tissue layer in focus (orange rays) will pass through the pinhole and will be detected. The majority of the fluorescence emitted from tissue layers out of focus (red and green rays) will be rejected. Illumination and collection therefore occur in the same focal plane (ie, they are confocal). Figure adapted with permission from Kumar S. *Development of Multidimensional Fluorescence Imaging Technology with a View towards the Imaging of Signalling at the Immunological Synapse* [doctoral thesis]. London: Chemical Biology Centre, Department of Chemistry, Imperial College London; 2010.^151^ **Abbreviation:** PMT, photomultiplier tube.

In confocal microscopy, the light source, the sample plane, and the detector are all confocal (ie, in conjugate image planes) (Figure 6). The illumination light is focused to a point in the sample, and all the fluorescence collected by the objective lens from that point is forced through a pinhole to reach the detector. Although other parts of the sample are also illuminated, light from out-of-focus planes in the sample is blocked by the pinhole and does not reach the detector. Thus, the main advantage of a confocal microscope is its optical sectioning ability, giving insight into the three-dimensional tissue structure and providing a virtual real-time histological diagnosis.

CLE is one of the newest advancements in diagnostic endoscopy and is a highly promising technique for investigating the mucosal surface together with its immediate subsurface areas. Cell structures and tissue morphological characteristics can be visualized to a maximum depth of 250 μm.

As mentioned above, two types of confocal endomicroscope have been developed: the confocal laser endomicroscope (eCLE) (Pentax Corporation), in which a miniaturized confocal scanner has been integrated into the distal tip of a conventional endoscope, and the probe-based system (pCLE) (Cellvizio^®^; Mauna Kea Technology, France), which can be passed through the working channel of standard endoscopes and has an external laser scanning unit.

#### eCLE

The confocal laser endomicroscope is based on micro-electro-mechanical system scanning mirror technology. The micro-electro-mechanical system scanning mirror oscillates to provide a scan pattern to the beam of light. A single-mode (Gaussian beam profile) fiber, acting as both the illumination point source and the detection pinhole, and a miniature objective lens at the distal end of a conventional video endoscope (EG3870K, EC-3870CILK; Pentax, Hoya Corporation, Japan) enable confocal microscopy in addition to standard video endoscopy.

A blue laser integrated into the endoscope light source is coupled into a single-mode fiber that delivers an excitation wavelength of 488 nm to the sample, and fluorescence emitted by the sample is detected at wavelengths above 505 nm. Viewing of a specific tissue depth is enabled by scanning successive points within the tissue in a grid of pixels along the x- and y-axes (parallel to the tissue surface) to produce sections of 475 μm ×475 μm (FOV) at variable imaging depths (range 0–250 μm). The lateral resolution is 0.7 μm, and the axial resolution is 7 μm. eCLE can produce conventional white-light endoscopic images and confocal images at the same time, with a pixel density of 1,024 pixels ×512 pixels at an acquisition rate of 1.6 frames per second or 1 ,024 pixels × 1,024 pixels at 0.8 frames per second.^94^,^95^

The distal tip of the endoscope contains an air- and water-jet nozzle, two light guides, an accessory channel used for irrigation and topical application of the contrast agent, and a 2.8 mm operative channel. Actuation of imaging plane depth is controlled using two remote control switches located on the handle.^26^

#### pCLE

pCLE consists of a coherent single mode fiber bundle, a miniature microscope objective, and two scanning mirrors at the proximal end of the bundle. The bundle has an outer diameter of 2 mm and can be introduced through the working channel of most endoscopes. In pCLE, a laser is sequentially focused onto single fibers at the proximal end of the bundle. The light exiting the fiber at the distal end is focused to a point in the sample using a miniature objective. Confocal fluorescence is focused by the objective onto the same fiber in the bundle. The light is detected on a detector, and the beam is eventually scanned over all fibers in the bundle to build up an image. The lateral and axial resolution range from 2.5 to 5.0 μm and from 15 to 20 μm, respectively.^96^ pCLE has a fixed image plane depth varying between 55 and 130 μm, depending on the probe used.^94^

#### Comparison of eCLE and pCLE

The main advantage of eCLE over pCLE is that the imaging plane depth is user tunable because the laser scanner is integrated into the endoscope and allows optical sectioning of the tissue at higher axial resolution (lateral resolution 0.7 μm, axial resolution 7 μm versus 2.5 and 15 μm, respectively), whereas in pCLE the imaging plane depth is fixed and cannot be adjusted.^95^–^97^

In pCLE, the resolution is limited by the number of fibers (30,000), but the image acquisition is faster than that of eCLE (12 versus 0.8–1.6 frames per second). One disadvantage of eCLE is the insertion tube diameter of the distal tip (12.8 mm) compared with the standard outer diameters of conventional endoscopes (9.0–11.1 mm). In contrast, pCLE, due to the small diameter of the bundle, permits the imaging of narrowed lumina such as the biliary and pancreatic ducts, ureters, and neoplastic or inflammatory strictures. Disadvantages of pCLE include limited lifespan (20 procedures), higher maintenance cost,^95^ and occupation of the endoscope working channel.

#### Problems with current CLE systems

A general drawback of the CLE technique is that intravenous (eg, fluorescein sodium) and topical (eg, acriflavine) fluorescence agents are used to achieve contrast. The avoidance of contrast agents would reduce both procedural time and any potential for associated adverse effects.

Fluorescein diffuses through the extracellular matrix of the epithelium and the lamina propria but does not stain cell nuclei.^98^ Acriflavine is a carcinogenic dye,^99^ and this clearly limits its clinical utility.^95^

Another drawback is the duration of the procedure, which can be almost twice as long as conventional endoscopy. In a study of contrast dynamics in porcine models, it has been reported that the best contrast and image quality can be achieved within the first 8 minutes after intravenous injection of 1% fluorescein, with highest signal-to-noise ratio (SNR) after 5 minutes. The SNR decreases significantly after 8 minutes.^100^ Furthermore, confocal endomicroscopy does not yet provide functional information about the tissue and is an examiner-dependent technique. Interobserver and intraobserver variability has not been adequately studied,^101^ and the interpretation of the intensity images is often somewhat challenging among users, who may require substantial supplementary training in histology or the presence of the pathologist in the endoscopy room to achieve acceptable diagnostic accuracy.^102^,^103^

#### Clinical experience with eCLE

##### Upper GI tract

Kiesslich et al^104^ proved that the overall accuracy for eCLE in predicting BE and associated neoplasia was 96.8% and 97.4%, respectively.

##### Lower GI tract

In 2004, Kiesslich et al^26^ showed that colonic dysplasia is detected using eCLE, with a sensitivity of 97.4% and a specificity of 99.4%. Detection by eCLE of dysplasia in Peutz–Jeghers syndrome has also been reported.^105^

The potential of eCLE to differentiate colorectal lesions was recently demonstrated by Sanduleanu et al.^106^ In this study, low-grade dysplasia was distinguished from HGD with high accuracy (96.7%), and eCLE predicted the final histology for all cases, with 95.7% accuracy.

#### Clinical experience with pCLE

##### Upper GI tract

In a prospective, double-blind review of 20 pCLE images of 40 sites of BE tissue by using matching biopsies as the reference standard, the preliminary evaluation accuracy and interobserver agreement of pCLE was assessed among eleven experts in BE imaging from four different endoscopy centers from the United States and Europe.^30^ Although the study was limited by the small sample size, high preliminary accuracy in diagnosis of BE dysplasia and neoplasia was achieved, with both inexperienced (90%) and experienced observers (95%). The main strength of the present study relative to other published data is the inclusion of a large group of endoscopists, only four of whom had previous experience with pCLE. Their high performance demonstrated that rapid training in image interpretation is feasible.

The first international multicenter, randomized, controlled trial using pCLE in 101 patients with BE was published in 2011. The sensitivity and specificity of pCLE in addition to HDE was compared with HDE alone for the detection of HGD and adenocarcinoma.^32^ Use of pCLE with HDE showed increased “per-location” sensitivity for the detection of both conditions compared with HDE alone (68.3% versus 34.2%). Instead, ‘per-location’ specificity was slightly reduced (87.8% versus 92.7%). Nevertheless, no statistically significant differences in “per-patient” sensitivity were observed between HDE, NBI, or pCLE. However, a high negative predictive value was collectively observed, and authors speculated that pCLE might facilitate early rule-out of dysplasia with high degree of confidence, allowing better informed decisions to be made for the management and subsequent treatment of patients with BE.

In a study by Bajbouj et al,^31^ optical biopsy with pCLE was compared with standard biopsy in the endoscopic evaluation of 68 patients (670 pairs of biopsies) with BE. Confocal images were interpreted live during examination as well as in a blinded controlled manner 3 months post-endoscopy, and findings compared with histology. pCLE was found to be comparable to endoscopic biopsy in excluding neoplasia, but due to its low positive predictive value and sensitivity, authors concluded that pCLE may currently not replace the standard practice for the diagnosis of BE-associated neoplasia.

##### Biliary and pancreatic ducts

Current techniques to detect malignant biliary and pancreatic strictures are of low sensitivity. The delay in tissue confirmation of malignancy can place a patient at risk for progression of disease precluding surgical resection. On the other hand, the inability to confirm whether a stricture is benign or malignant could lead to unnecessary surgery.

In 2008, Meining et al^107^ used pCLE to examine the biliary tract of 14 patients with biliary strictures at the liver hilus or the common bile duct. Patterns indicative of neoplasia were identified, and pCLE predicted neoplasia with an overall accuracy of 86%, surprisingly outperforming preoperative tissue sampling (79%). After surgery, histopathology revealed invasive adenocarcinoma in two patients in which preoperative biopsy specimens were negative for neoplasia, whereas pCLE indicated the presence of malignancy. Also the median SNR derived from a region of interest in both normal and neoplastic epithelium was significantly different (lower in neoplasia), accounting for less broad intrinsic fluorescence intensity distribution in neoplastic tissue, which reflects different concentration of fluorophores following structural neoplastic rearrangement. A dark gray background with poor mucosal structure and large white streaks (vessels) was mainly seen in neoplastic strictures. On the contrary, a reticular pattern of different gray scales and villous structure without white streaks was seen in patients without neoplasia.

Peroral pancreatoscopy and pCLE has also been reported by the same group in a case of intraductal papillary mucinous neoplasm (IPMN).^108^ IPMN often goes unrecognized in patients with chronic pancreatitis, and its diagnosis is rather challenging. Based on the findings obtained with these two imaging methods, the diagnosis of IPMN was formed and was then confirmed by histology.

Loeser et al^109^ used pCLE in 14 patients with indeterminate biliary strictures together with standard biopsies and brushings. In parallel, they also examined rat bile ducts ex vivo using multiphoton microscopy to better understand the nature of the human bile duct structures visualized during confocal endomicroscopy. Of the 14 patients, six had a diagnosis of cancer. None of the criteria used to evaluate possible malignancy in the confocal images was found to be sufficiently specific for malignancy. An abnormal reticular network, which may reflect changes in lymphatic vessels, was never seen in benign strictures, and the authors proposed that in the absence of markers of potential malignancy, if a normal reticular pattern is visible then the diagnosis of malignancy is unlikely. The reticular pattern seen in normal tissue was believed to be a network of lymphatic vessels, as shown in multiphoton reconstructions of intact rat bile ducts and confirmed by special stains.

In an international multicenter study involving 102 patients with indeterminate pancreatobiliary strictures, pCLE outperformed tissue sampling in sensitivity (98% versus 45%), negative predictive value (97% versus 69%), and overall accuracy (81% versus 75%). However, pathology was unbeatable in specificity and positive predictive value. Delivery of the confocal miniprobe through a cholangioscope or via a catheter did not lead to significant differences in sensitivity and specificity.^33^

##### Lower GI tract

Confocal imaging of a DALM in a patient with chronic ulcerative colitis has also been reported.^110^ Morphological characterization with pCLE was performed both over the lesion and adjacent inflamed mucosa, showing features suggestive of dysplasia and inflammation, respectively. The main aspect of inflamed mucosa consisted of dilation of crypt openings, irregular arrangement of crypts, crypt destruction and fusion, and crypt abscess. Dark mucin-depleted goblet cells and villiform epithelial digitations were recognized as dysplastic features in agreement with the Mainz classification for prediction of intraepithelial neoplasia.^26^

A prospective pilot study on 22 patients under surveillance for ulcerative colitis was conducted by van den Broek et al^113^ using NBI plus HDE followed by pCLE before taking targeted and random biopsies. Reasonable diagnostic accuracy was achieved, although movement artifacts significantly impaired the video quality.

Considering histology as gold standard, Buchner and Wallace^112^ demonstrated that pCLE can accurately discriminate between hyperplastic and adenomatous polyps, and detect residual adenomatous tissue after EMR. They then compared pCLE with NBI and FICE, collectively named as virtual chromoendoscopy, in 119 polyps (81 neoplastic and 38 hyperplastic) from 75 patients.^113^ pCLE had higher sensitivity compared with virtual chromoendoscopy (91% versus 77%) for classification of colorectal polyps using histopathology as gold standard. In that study, HDE was used as the primary inspective technique. Prior to pCLE imaging, either FICE or NBI, depending on availability, were used after detection of a suspicious lesion. They also examined the learning curve faced by clinicians in correctly identifying benign and neoplastic colorectal lesions by using pCLE.^114^ Accuracy of image interpretation and acquisition increases with the number of images observed (up to 86% beyond 60 lesions).

In a recent prospective multicenter study, 92 patients who had their lesion removed by EMR in a previous colonoscopy were referred for follow-up colonoscopy within 1 year and inspection of EMR scars (n=129) with NBI or FICE and pCLE to detect residual neoplasia. Accuracy of pCLE alone and in combination with NBI or FICE against histopathology as the gold standard was of 81% and 90%, respectively; whereas the cumulative accuracy of virtual chromoendoscopy (NBI + FICE) alone was 77%.^34^

In vivo characterization of 32 superficial colorectal neoplastic lesions with pCLE was performed by De Palma et al^115^ in 20 consecutive patients. pCLE presumptive diagnoses were compared with histopathology of resected lesions or targeted biopsies. The sensitivity and specificity of pCLE to differentiate neoplastic from hyperplastic lesions were 100% and 84.6%, respectively.

The use of molecular biomarkers in combination with confocal endomicroscopy was first investigated by Hsiung et al^83^ to develop a fluorescent probe for detecting colon cancer. They identified a specific heptapeptide sequence, VRPMPLQ, which was conjugated with fluorescein and tested in patients undergoing colonoscopy. The fluorescein-conjugated peptide was administered topically and was found to bind more strongly to dysplastic cells than to adjacent normal cells, with 81% sensitivity and 82% specificity, respectively.

Wang et al^27^ conducted a pioneering observational study using pCLE in 54 patients undergoing bowel cancer screening colonoscopy, with the aim to track the uptake and distribution of fluorescein from the crypts to the lamina propria after topical administration. This contributed to further understanding the functional anatomy of the colonic glands in normal, hyperplastic, and adenomatous tissue in vivo. As with histology, the typical shape and size of glands for each tissue type is clearly discriminated, and there is correlation between tissue morphology and time of transit, with significantly longer time of passage through adenomatous mucosa (>5 seconds) compared with hyperplastic or normal tissue. High diagnostic accuracy is achieved using the speed of absorption of fluorescein (contrast ratio) as a discriminant function to distinguish normal from diseased mucosa (89%), hyperplasia from adenoma (96%), and even tubular from villous adenoma (93%).

Moderate to good interobserver agreement among three international experts and 76% accuracy in diagnosis of neoplasia was achieved using pCLE in a study on 53 patients with 75 colorectal lesions, 50 of which were neoplastic.^116^ The authors concluded that current accuracy and interobserver agreement do not yet support routine clinical use in screening or surveillance colonoscopy.

### EC

EC (Olympus Corporation) is a novel imaging technique, enabling microscopic imaging of the GI mucosal surface with a magnification of up to ×1,400.^28^ EC is based on a contact light microscope which enables real-time visualization of cellular structures of the superficial epithelial layer in a plane parallel to the mucosal surface. Cytological and architectural features, such as the size and shape of cells, nuclei, and the nucleus to cytoplasm ratio, can be assessed. The technique uses a fixed-focus, high-power objective lens that projects highly magnified images onto a CCD at a rate of 30 frames per second.^117^

As for CLE, two types of EC systems are currently available on the market. A probe-based handheld miniprobe (pEC), providing magnification of up to ×570 and ×1,400, which can be passed through the working channel of a conventional endoscope, and a system integrated into the distal tip of an endoscope (iEC), providing magnification of up to ×580.

FOVs are 300 μm ×300 μm, 120 μm ×120 μm, and 400 μm ×400 μm for ×570 pEC, ×1,400 pEC, and ×580 iEC, respectively. The axial resolution varies from 0–50 μm, and the lateral resolution from 1.7–4.0 μm.

Using both systems, contact with the tissue surface is necessary for imaging. EC requires preparation of the mucosal layer with absorptive contrast agents like methylene blue or toluidine blue.^28^ Prior to imaging and tissue staining the mucosal surface must be treated with a mucolytic to remove excess mucous. Repeat staining is often needed after ~5 minutes of imaging.

#### Clinical experience

Promising initial results, primarily in identifying and discriminating neoplastic from non-neoplastic tissue, have been shown in a few prospective studies.^117^–^120^ Notably, EC is able to detect dysplasia in aberrant crypt foci of normal colon mucosa surrounding cancer,^118^ and discriminate invasive colon cancers from adenomatous polyps.^120^ In contrast, only a limited role has been found in detecting and predicting early esophageal squamous cell carcinoma.^121^ The potential of EC for the in vivo characterization of duodenal mucosa in coeliac disease has also been reported.^122^

Nevertheless, there is currently a paucity of interest and research on EC, possibly due to the requirement of topical staining, the relative lack of axial discrimination, and the low resolution images compared with other microscopy techniques such as CLE.

## Future potential optical imaging modalities

Future prospects in endoscopic imaging include optical coherence tomography,^123^–^125^ multiphoton microscopy,^126^–^131^ second harmonic generation imaging,^131^,^132^ Raman endoscopy,^133^–^135^ coherent anti-Stokes Raman scattering microscopy,^136^–^138^ and FLIM.^139^–^142^

Studies with endoscopically compatible fiber-optic probes or prototype endoscopes are under way for validation of preliminary results. However, there is still a significant amount of technological development and clinical study required before they can become clinically viable methods.

## Conclusion

Recent advances in endoscopy constitute an unprecedented leap forward in basic and clinical research for both patients and clinicians. However, the full adoption of any of these techniques in clinical practice still requires further extensive evaluation. For instance, especially in patients with dysplasia and early cancers, the objectivity and inter-rater reliability for each of these techniques have not been well studied, and this clearly affects their still low perceived clinical utility and acceptance. Future work could be directed toward the integration of analytical techniques such as Raman spectroscopy^137^ or fluorescence lifetime spectroscopy^143^,^144^ in conventional accessory devices such as biopsy forceps or snares that could be operated in conjunction with advanced imaging techniques and potentially offer an objective complement to established wide-field image-enhanced techniques such as HD-WLE, NBI, or AFI, which still suffer from operator-subjectivity and poor specificity.^75^–^77^,^135^,^145^–^146^ In addition, these techniques can only infer structural aspects and do not provide functional information about the tissue. It is still unknown which approach or combination of techniques offers the best potential. The optimal method will probably entail the combination of a wide-field overview technique with an optical microscopy method. In this respect, the challenging combination of AFI and CLE seems promising, and further research is awaited. One optical technology potentially capable of this is FLIM.^147^ A FLIM scanning confocal endomicroscope design,^35^,^148^,^149^ and a wide-field FLIM endoscope probe^150^ have recently been proposed, and current efforts are directed to implement and validate this technology for modern endoscopy.
